# Association between Optical Coherence Tomography Measurements and Clinical Parameters in Idiopathic Intracranial Hypertension

**DOI:** 10.1155/2021/1401609

**Published:** 2021-01-25

**Authors:** Priscilla Fernandes Nogueira, Gustavo Coelho Caiado, Carolina P. B. Gracitelli, Fernando Meister Martins, Felipe Chaves Duarte Barros, Sandro Luis De Andrade Matas, Sérgio Henrique Teixeira, Luciana da Cruz Noia, Danilo Andriatti Paulo

**Affiliations:** ^1^Department of Ophthalmology and Visual Science, Federal University of São Paulo, Sena Madureira, São Paulo, Brazil; ^2^Centro de Estudos Alcides Hirai, Ver Mais Oftalmologia, Vinhedo, São Paulo, Brazil; ^3^Vera Cruz Oftalmologia, Hospital Vera Cruz, Campinas, SP, Brazil; ^4^Department of Neurology and Neurosurgery, Federal University of São Paulo, Sena Madureira, São Paulo, Brazil

## Abstract

**Purpose:**

To correlate optical coherence tomography (OCT) measurements with clinical parameters in idiopathic intracranial hypertension (IIH).

**Methods:**

A cross-sectional study was conducted with 22 patients with IIH and 11 controls. All participants underwent comprehensive ophthalmological examination followed by spectral-domain OCT (SD-OCT) and standard automated perimetry using the 30–2 program of the Humphrey visual field analyzer. Correlations between ganglion cell complex (GCC) thickness and retinal nerve fiber layer (RNFL) thickness, as measured by SD-OCT, and clinical parameters were assessed using generalized estimating equations.

**Result:**

The mean age of the participants was 35.0 ± 10.83 years. The groups were similar regarding age, but were significantly different regarding sex and visual acuity (*p*=0.001 and *p*=0.038, respectively). The GCC was significantly thinner in the IIH group, with a mean of 90.535 ± 9.766 *μ*m compared to 98.119 ± 6.988 *μ*m for the controls (*p*=0.023). There was a significant association between GCC thickness and optic disc pallor (*p*=0.016) and between edema and visual acuity (*p*=0.037). No significant difference was found in RNFL thickness between patients and controls.

**Conclusion:**

The GCC was thinner in the patients with IIH compared to the controls, and there was an association between GCC and optic disc pallor. This might suggest a role for OCT parameters when the structural changes that occur in IIH are investigated, possibly guiding clinical decision making.

## 1. Introduction

Idiopathic intracranial hypertension (IIH) is a clinical condition of uncertain etiology characterized by high intracranial pressure (ICP) in the absence of expansive or vascular lesions, enlargement of the cerebral ventricles, or focal neurological signs except for sixth nerve palsy [1, 2]. IIH usually affects overweight and obese women. Ocular and systemic symptoms are secondary to hypertension, which may course with or without papilledema, leading to blindness in up to 10% of cases [1–3]. The incidence of IIH is 0.9/100,000 people in the general population and 19.3/100,000 in individuals with typical IIH phenotype [4–6]. The natural history of IIH may vary. For many cases, it is a self-limiting condition, while in other cases, intracranial pressure remains elevated for several years [7]. Its chronicity may be associated with optic atrophy, resulting in irreversible visual impairment and defects [7]. The main symptoms of IIH include headache (68–98%), tinnitus (60%), transient visual obscurations (70%), and diplopia (40%), with papilledema as its main sign. However, some patients may be asymptomatic [5–7].

Papilledema refers to optic disc swelling caused by intracranial hypertension. In general, it is bilateral, but may be asymmetrical. Papilledema probably results from increased perineural pressure with subsequent axonal swelling in the optic nerve head [5]. A correlation between papilledema and visual prognosis has been reported, particularly when loss of macular ganglion cells implies a poor visual prognosis [6, 8, 9]. Chronic swelling leads to progressive optic nerve pallor, mainly due to the loss of nerve fibers and ganglion cell axons, resulting in irreversible blindness [1, 2].

Since use of the Frisén classification in IIH for rating the severity of papilledema involves some limitations [3], further research on a better quantitative approach is required to investigate the prognosis of the disease. Accordingly, optical coherence tomography (OCT), a simple noninvasive tool that is sensitive enough to assess the peripapillary retina, retinal nerve fiber layer (RNFL), macula, and the prelaminar optic nerve, has been proposed for this purpose [3, 5, 6]. In the retina, OCT images may reveal abnormalities in the papillomacular bundle area in cases of acute and chronic IIH [6]. Such abnormalities may refer to reduced RNFL thickness in patients with chronic IIH or increased RNFL thickness in patients with acute IIH and swelling. The visual prognosis of patients with optic nerve swelling and increased RNFL thickness in the macular and peripapillary region over longer periods of time may be poorer [6, 9–11]. Identifying structural damage to the optic nerve and RNFL constitutes an essential step in diagnosing and managing IIH. The introduction of spectral-domain OCT (SD-OCT) has allowed damage to these structures to be objectively quantified with unprecedented resolution. In addition, recent attention has been focused on performing imaging tests on the macular area to quantify the loss of neural tissue caused by the disease (18 [12]). OCT en face ins another option which is a valid clinical tool for monitoring papilledema in IIH, but is typically used to determine measurements of the peripapillary retinal nerve fiber layer [13].

Depending on the specific parameter to be evaluated and the characteristics of the study population, sensitivity for detecting damage according to the best performing RNFL parameters has been reported to range from approximately 60% to 98%, with specificity ranging from 80% to 95%. In fact, although sectoral RNFL parameters may increase the likelihood of detecting localized RNFL damage, the reproducibility of these parameters is often poor, as mean measurements refer to only relatively small areas. On the other hand, mean global RNFL thickness has generally been shown to be the most reproducible parameter, which is not surprising considering that its calculation involves mean measurements over a relatively large area. The gain in reproducibility, however, may come at the potential cost of missing some localized RNFL defects, particularly in cross-sectional evaluations. Nonetheless, even for the detection of localized RNFL defects, global RNFL thickness measurements still appear to perform at least as well as sectoral parameters, suggesting that the improved reproducibility may overcome the limitation of using mean measurements taken over a large area. In recent years, attention has increasingly been directed towards the macular region for the evaluation of glaucomatous damage. As a large proportion of total macular thickness is composed of RNFL and ganglion cell bodies, this region is an attractive area to identify structural damage caused by the disease. The macular retinal ganglion cell layer contains more than 50% of the ganglion cells of the entire retina (18). However, to the best of our knowledge, no study has yet been conducted in which macular and peripapillary ganglion cells are investigated in patients with IIH and then correlated with clinical findings.

Therefore, the purpose of the present study was to evaluate the correlation between OCT assessments of RNFL thickness and ganglion cell complex (GCC) thickness and the clinical findings in patients diagnosed with IIH.

## 2. Methods

This cross-sectional study was conducted in compliance with the requirements laid out in the Declaration of Helsinki. The internal review board of the Federal University of São Paulo approved the study protocol under reference UNIFESP/EPM-CEP 2418003. All the participants signed an informed consent form prior to their inclusion in the study. The complete Excel database generated in this study is provided in the supplementary data file to support the present findings.

### 2.1. Study Participants

A total of 22 patients with IIH and 11 controls were included in the study. A review of their medical history was conducted, and all the participants underwent a comprehensive ophthalmologic examination that included a visual acuity test, slit-lamp biomicroscopy, intraocular pressure measurement (Goldmann tonometer), gonioscopy with Possner goniolens, and dilated fundoscopic examination with a 78-diopter lens. In addition, the participants were submitted to standard automated perimetry (SAP) using the 30–2 program of the Humphrey visual field analyzer (Carl Zeiss Meditec, Inc, Dublin, CA) and underwent OCT according to the methodology described below.

Participants in the IIH group had to be between 18 and 60 years of age with signs and symptoms of generalized intracranial hypertension or papilledema, documented elevated intracranial pressure with an opening pressure ≥25 cmH2O (measured by lumbar puncture with the patient in the lateral decubitus position with legs extended and head and back strictly horizontal); normal cerebrospinal fluid (CSF) composition; no evidence of hydrocephalus, tumor mass, or vascular injury at magnetic resonance imaging (MRI) or computed tomography (CT), and normal neurological examination except for sixth cranial nerve palsy.

The participants of the control group were selected from the general community. Controls had to be between 18 and 60 years of age, with a corrected visual acuity ≥20/40, no ocular or systemic disease that could affect the visual field or alter the optic nerve, RNFL, or GCC; absence of opaque media; a current intraocular pressure <21 mmHg, and no previous history of elevated intraocular pressure, intraocular infection, eye surgery, or trauma. In addition, controls should have a normal optic disc.

The exclusion criteria for both groups were age <18 years or >60 years, any concomitant physical or psychiatric diseases, other eye diseases, and refusal to sign the informed consent form. Individuals with coexisting retinal disease or nonglaucomatous optic disc neuropathy and those for whom the spherical equivalent was 5.0 diopters or greater were also excluded from the study.

### 2.2. Visual Field Analysis

The exams conducted with standard automated perimetry were performed in a dark room with 1-lux ambient lighting level. While one eye was tested, the other was occluded.

The perimeter used was the Humphrey Field Analyzer II (Carl Zeiss Meditec, Dublin, California, USA) with the Swedish Interactive Threshold Algorithm-Standard (SITA-Standard). The assessment was performed in the central 30° of the patient's visual field, with a Goldmann size III white light stimulus. Only participants with reliable tests were included in the study. The test reliability applied the following criteria: loss of fixation ≤20%, and false positives and false negatives ≤15%. Visual fields with the following artifacts were excluded: evidence of border and eyelid artifacts, effects of inattention or fatigue, or damage to the visual field caused by a disease other than IIH.

### 2.3. OCT Evaluation

The RNFL of the macular, peripapillary, and papillary regions was evaluated by spectral-domain OCT (Optovue RTVue-100, Fremont, CA, USA). The RTVue-100 software program, version 4.0, was used for data analysis. The GCC measurements consisted of one horizontal line scan of 7 mm in length (467 A-scans) and 15 vertical line scans of 7 mm in length (each consisting of 400 A-scans) taken at 0.5 mm intervals. The scan center was shifted 0.75 mm towards the temporal periphery and encompassed a rectangular area measuring 7 × 7 mm. This scan configuration provided 14,810 A-scans in 0.58 s. The GCC thickness was defined as the distance between the inner limiting membrane (ILM) and the outer border of the inner plexiform layer (IPL). The nerve fiber layer (*μ*m), the ganglion cell layer (*μ*m), and the internal plexiform layer (*μ*m) were evaluated. The peripapillary RNFL was evaluated using the fast RNFL thickness protocol. The fast RNFL scans consisted of three peripapillary scans (256 A-scans each) with a diameter of 3.4 mm, calculated by estimating the distance between the ILM and Bruch's membrane in a cylinder of 6 mm in diameter using the segmentation algorithm contained in the device software. The 3D optic nerve head (ONH) scan was performed using a custom protocol with 145 slices (B-scans), focusing the ONH with a scanning angle of 15° × 15° and a resolution of 384 A-scans per B-scan. The best peripapillary scan, based on a best quality signal for each eye, was selected for further analysis. When high-quality images, defined as a signal strength index of 30 or more, were not obtained, the individuals were excluded from the analysis. The OCT was performed within 1 week of CSF puncture in the patients with IIH.

### 2.4. Statistical Analysis

Descriptive statistics included the calculation of means and standard deviations (SD) and medians and interquartile ranges for variables with normal and nonnormal distribution, respectively. The Skewness-Kurtosis test was used to determine whether or not distribution was normal. The *t*-test was used to perform multiple comparisons between the groups (IIH and controls), or in the case of nonparametrically distributed variables, the Wilcoxon rank-sum test was used. Fisher's exact test was used for the categorical variables.

When eligible, both eyes of each participant were included in this analysis. To correct for the bias introduced by the expected correlation between the two eyes of each participant, a generalized estimating equation (GEE) was used to adjust for intereye correlations [14]. Next, a GEE was used to examine the relationship between the GCC or RNFL thickness (dependent variables) and the clinical findings such as opening pressure, use of the carbonic anhydrase inhibitor, acetazolamide, CSF diversion procedures and optic nerve sheath fenestration, edema, pallor, and visual acuity. Whenever significant associations were found, age and intraocular pressure were evaluated as covariants.

Statistical analyses were performed using the commercially available Stata software program (version 13, StataCorp LP, College Station, TX, USA). The *α* level (type I error) was set at 0.05.

## 3. Results

A total of 22 patients with IIH (43 eyes) and 11 controls (22 eyes) were included in the present study. There was no statistically significant difference between the two groups with respect to age, with the mean age of the participants in the IIH group being 34.90 ± 12.06 years compared to a mean of 35.45 ± 8.61 years for those in the control group (*p*=0.809). Differences were found between the two groups with respect to sex (*p*=0.001) and visual acuity (*p*=0.038).  Table 1 summarizes the demographic characteristics of the participants and the clinical findings.

In the IIH group, the mean opening pressure was 25.714 ± 6.827 cmH2O (range 16 to 45 cmH2O). The optic disc head was normal in 30 eyes (69.77%), while optic disc swelling was found in 25 eyes (58.14%), pallor on fundoscopy was found in 6 eyes (13.95%), and central nervous system venous thrombosis was found in 7 patients (31.82%). In the entire sample, 8 patients (36.26%) were in use of acetazolamide 250 mg (2 tablets in the morning and 1 tablet at night). None of the patients in this study were candidates for bariatric surgery, CSF diversion procedures, or optic nerve sheath fenestration.

**Figure 1:**
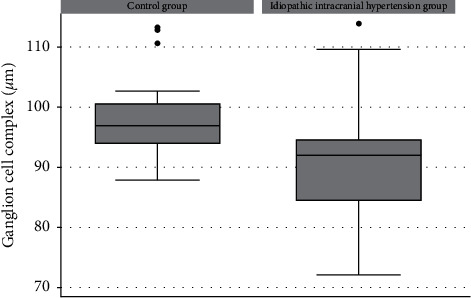
: Boxplots showing the distribution of the ganglion cell complex in the control and IIH groups. Box: median and interquartile range. Boxplot with whiskers with a maximum and minimum 1.5 IQR.

There was no statistically significant difference in RNFL thickness between the participants in the IIH group and those in the control group (111.637 ± 24.941 *μ*m and 116.012 ± 110.1010 *μ*m, respectively; *p*=0.453). However, the GCC was thinner in the IIH group compared to the control group (90.535 ± 9.766 *μ*m and 98.119 ± 6.988 *μ*m, respectively; *p*=0.023, Table 2).  Figure 1 shows the distribution of GCC values in both groups.

An association was identified between ocular findings and different clinical and demographic parameters for the entire IIH group. A significant correlation was found between GCC thickness and pallor (*p*=0.016). In addition, a significant correlation was observed between swelling and visual acuity (*p*=0.037). Following adjustment for age and intraocular pressure, the association between thinner GCC measurements and pallor and the association between edema and visual acuity persisted (*p*=0.028 and *p*=0.036, respectively).

**Figure 2:**
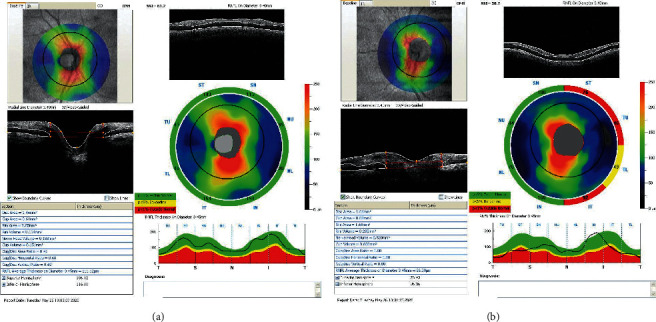
: Examples of OCT measurements of IIH and control patient matched by age. (a) Right eye RNFL measurement of an individual of the control group, 29-year-old patient, with RNFL average thickness on diameter 3.45 mm = 111.12 *μ*m. (b) Left eye RNFL measurement of an IIH 53-year-old patient, with RNFL average thickness on diameter 3.45 mm = 86.39 *μ*m.

**Figure 3:**
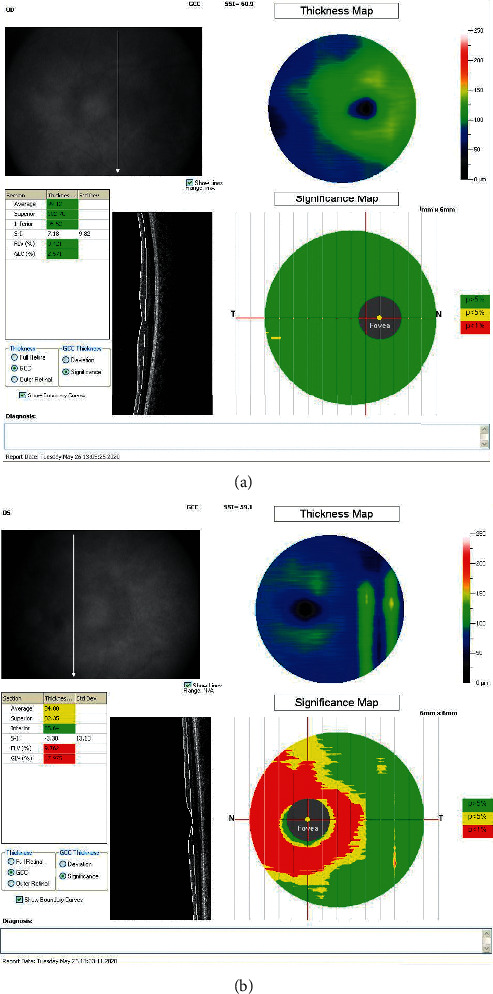
: Examples of OCT measurements of IIH and control patient matched by age. (a) Right eye GCC measurement of an individual of the control group, 29-year-old patient, with a GCC average thickness 99.12. (b) Left eye GCC measurement of an IIH, 53-year-old patient, with GCC average thickness 84.00.

Figures 2 and 3 illustrate two examples of printouts of the RNFL and GCC, respectively, in IIH and control patients.

In addition, all data are available in the supplementary data.

## 4. Discussion

This cross-sectional study was conducted to evaluate GCC and RNFL thickness, as determined by SD-OCT, in patients with IIH and controls. The patients with IIH were found to have thinner GCC compared to controls. In addition, the thickness of the GCC was associated with optic nerve pallor. To the best of our knowledge, this is the first study in which OCT was used to identify an association between GCC and visual acuity in patients with IIH.

A retrospective study proposed by Padhye et al. who applied the same idea of correlating imaging results with visual parameters; however, those investigators used neuroimaging. In that study, the medical records of 35 patients diagnosed with IIH were reviewed and parameters of visual function were correlated with orbital MRI findings. No significant correlations were found, suggesting that MRI parameters may not represent valid surrogate markers for loss of vision in patients with IIH. Although MRI parameters can help identify the presence of papilledema, they cannot predict results and are not of use in monitoring treatment [6].

As in the present study, Kaufhold et al. also used SD-OCT. However, those investigators focused on optic nerve head (ONH) volume as a potential marker of the effectiveness of treatment and disease progression in IIH. In that cross-sectional pilot study in which 19 patients with IIH were compared with 19 controls, a significant correlation was found, with the volume of the ONH calculated using SD-OCT serving as a parameter for diagnosis of and progression in IIH. In fact, ONH volume ultimately proved more sensitive than the measurement of intracranial pressure [10].

Previous studies evaluated the role of GCC or RNFL thickness in cases of IIH with papilledema [4, 7, 9, 10, 15]. OCT measurements will generally be abnormal in patients with the active disease, with increases in the neuroretinal rim thickness and in the optic disc area and a decrease in the volume of the physiologic excavation in all grades of papilledema [16]. There must be an increase in RNFL thickness during the acute phase of papilledema; conversely, there should be less of an increase or no increase at all in RNFL thickness in patients with optic atrophy, while thickness should be normal in the patients in remission from the disease without sequelae [16] and in healthy controls. The ganglion cell layer is generally thinner in other forms of papilledema such as that found in cases of multiple sclerosis [17] and ischemic optic neuropathies [18], leading to a poor prognosis insofar as vision is concerned. Although GCC is generally expected to be normal in patients with IIH, patients with abnormalities are expected to progress to optic atrophy and decreased visual acuity [19]. In the present study, the objective was to evaluate GCC thickness in patients with IIH using the minimally invasive and highly sensitive SD-OCT exam and to correlate findings with visual acuity. Since our results were statistically significant, we consider that useful information has been obtained that will help manage the patient with IIH. It may, therefore, be possible to avoid invasive and unpleasant tests such as lumbar puncture for the assessment of CSF pressure to control the progression of IIH.

The principal objectives of this study were to investigate the thickness of the macular GCC, as evaluated by OCT, and to correlate the results with optic nerve pallor and visual prognosis in patients with IIH compared to a control group. The results should help assure adequate control for all patients with IIH, avoiding visual sequelae or irreversible loss of vision.

Some limitations of the present study must be taken into consideration. (1) This was a cross-sectional study, and the sample size was small; however, it is the first study to identify a correlation between GCC and optic disc pallor. (2) The clinical examinations were subjective; however, one single ophthalmologist performed all the examinations to avoid any possible bias. (3) The usefulness of OCT in predicting poorer visual acuity in patients with IIH has its limitations. Although papilledema is a characteristic sign of intracranial hypertension, it has been reported to be absent in 6% of IIH patients [9]. Therefore, patients must also be followed up with lumbar puncture and visual field testing to manage control and monitor progression of the disease. (4) Patients with any systemic disease and those in use of any medication that could affect GCC or RNFL thickness were excluded from this sample; however, there are unknown factors such as eyelid position or unreported medications that could also have affected these measurements. These factors could have caused a bias in the results of both groups. (5) Future studies using a longitudinal approach will be necessary to investigate the usefulness of OCT measurements in the management of IIH.

In conclusion, there are significant correlations between the thickness of the GCC and optic disc pallor and between the presence of papilledema and poor visual acuity in patients with IIH. These results could possibly be extrapolated to other diseases that present with papilledema and may be useful in the future to ensure better control and treatment, hence preventing progression of the disease and loss of vision.

## Figures and Tables

**Table 1:** Demographic and clinical characteristics of study patients (mean ± standard deviation).

	Control subjects (*N* = 11 subjects, 22 eyes)	IIH patients (*N* = 22 subjects, 43 eyes)	*p* value
Age (years)	35.45 ± 8.61	34.90 ± 12.06	0.809
Sex, female (%)^*∗*^	8 (17,02%)	39 (82,9%)	0.001
SAP VFI	NA	89.417 ± 24.898	NA
SAP PSD (dB)	NA	3.054 ± 2.302	NA
SAP MD (dB)	0.0 ± 0.8	−6.9 ± 7.3	<0.001
Visual acuity (LogMAR)	90.7 ± 10.8	68.3 ± 12.0	0.038
Mean opening pressure (cmH2O)	NA	25.714 ± 6.827	NA
Visual acuity (LogMAR)	0.00 ± 0.00	0.02 ± 0.11	0.038
Intraocular pressure (mmHg)	NA	14.757 ± 3.278	NA
Paquimetry (*μ*m)	NA	561.118 ± 62.949	NA

^*∗*^Fisher's exact test. *t*-test. SAP = standard automatic perimetry; MD = mean deviation; dB = decibels; PSD = pattern standard deviation; VFI = visual field index.

**Table 2:** . OCT parameters of study patients (mean ± standard deviation).

	Control subjects (*N* = 11 subjects)	IIH patients (*N* = 22 subjects)	*p* value
RNFL thickness (*μ*m)	116.012 ± 110.101	111.637 ± 24.941	0.453
GCC thickness (*μ*m)	98.119 ± 6.988	90.535 ± 9.766	0.023

^*∗*^ *t*-test. IIH = idiopathic intracranial hypertension; RNFL = retinal nerve fiber layer; GCC = ganglion cell complex.

## Data Availability

All data are available in the supplementary data.
